# Cracking Open Bacterial Membrane Vesicles

**DOI:** 10.3389/fmicb.2019.03026

**Published:** 2020-01-17

**Authors:** Toshiki Nagakubo, Nobuhiko Nomura, Masanori Toyofuku

**Affiliations:** ^1^Department of Life and Environmental Sciences, University of Tsukuba, Tsukuba, Japan; ^2^Microbiology Research Center for Sustainability, University of Tsukuba, Tsukuba, Japan

**Keywords:** membrane vesicles, proteome, lipidome, cargo selection, endocytosis, membrane fusion

## Abstract

Membrane vesicles (MVs) are nanoparticles composed of lipid membranes that are produced by both Gram-negative and Gram-positive bacteria. MVs have been assigned diverse biological functions, and they show great potential for applications in various fields. However, the mechanisms underlying their functions and biogenesis are not completely understood. Accumulating evidence shows that MVs are heterogenous, and different types of MVs with different compositions are released from the same species. To understand the origin and function of these MVs, determining the biochemical properties of MVs is important. In this review, we will discuss recent progress in understanding the biochemical composition and properties of MVs.

## Introduction

Membrane vesicles (MVs), which are produced by most bacteria, have diverse biological functions. These functions are not only interesting from a biological perspective but also for their great potential for broad applications in immunology and biotechnology.

Membrane vesicles consist of various types of lipids derived from cellular membranes along with numerous other biomolecules, such as membrane, periplasmic, and cytoplasmic proteins; DNA; RNA; and low molecular mass organic compounds that confer various biological functions (Brown et al., 2015; Schwechheimer and Kuehn, 2015; Dauros-Singorenko et al., 2018; Toyofuku et al., 2019). Recent studies have shown that there are different pathways of MV biogenesis, which produce different types of MVs (Toyofuku et al., 2019). Thus, the mechanism of MV formation determines its biochemical composition. Knowledge of the composition of MVs is important for understanding both the mechanisms of biogenesis and their biological functions. A number of studies have examined the biochemical properties of MVs, and in this review, we will summarize the recent progress in understanding the basic properties of MVs.

## Composition and Biogenesis of Bacterial MVs

In the following sections, we summarize biochemical analyses of bacterial MVs that provided insights into their biogenesis.

### Protein Composition of MVs in Gram-Negative Bacteria

**FIGURE 1:**
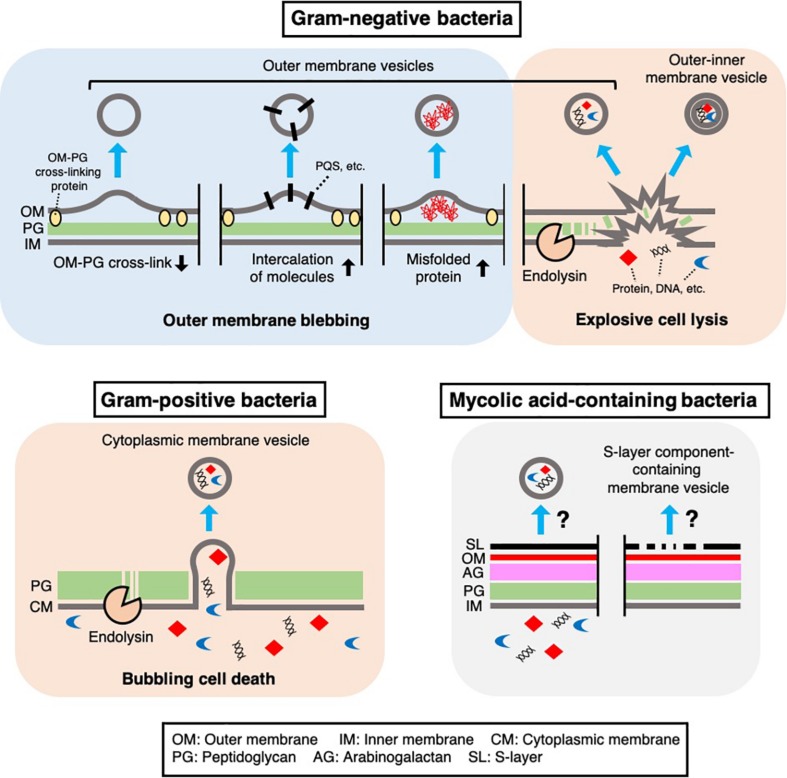
Mechanisms of MV biogenesis. Several mechanisms underlying MV formation in bacteria have been proposed. In Gram-negative bacteria, outer membrane vesicles and outer-inner membrane vesicles are produced through outer membrane blebbing or explosive cell lysis. Outer membrane blebbing is induced by structural changes in the cell envelope, such as a decrease in outer membrane-peptidoglycan cross-linking proteins, intercalation of molecules [such as *Pseudomonas* Quinolone Signal (PQS)] in the membrane, or the accumulation of misfolded proteins at specific regions of the cell envelope. Explosive cell lysis is triggered by phage-derived endolysin, which degrades the cell wall. In Gram-positive bacteria, cytoplasmic membrane vesicles are produced through bubbling cell death, in which phage-derived endolysin degrades the cell wall and the cytoplasmic membrane protrudes through the resulting holes in the peptidoglycan. In mycolic acid-containing bacteria, the mechanism of MV formation remains unknown, although there is evidence that these bacteria produce MVs containing inner membrane lipids or cell envelope associated-proteins such as S-layer component proteins.

Classical MVs are often referred as outer membrane vesicles (OMVs), which are generated through blebbing of the outer membrane in Gram-negative bacteria (Schwechheimer and Kuehn, 2015; Jan, 2017). Hoekstra et al. (1976) reported that MVs from <volume>*Escherichia coli* are derived from the outer membrane. In this study, they demonstrated that the lipid composition, SDS-PAGE protein profile, and specific activities of several membrane enzymes in these MVs are similar to those in the outer membrane (Hoekstra et al., 1976). This pioneering study showed that some proteins, including lipoproteins, were less abundant in OMVs than in the cellular outer membrane, suggesting that OMVs may originate from specific outer membrane regions (Hoekstra et al., 1976). Lpp is the most abundant lipoprotein in *E. coli*, and its lipid moiety is anchored to the outer membrane (Schwechheimer et al., 2013). Lpp exists in both “free” and “bound” forms, which are respectively outer membrane-anchored or covalently cross-linked to peptidoglycan through a linkage between the outer membrane and peptidoglycan layer (Schwechheimer et al., 2013). The cross-link between Lpp and peptidoglycan is formed at the C-terminal lysine by L,D-transpeptidases in *E. coli* (Magnet et al., 2007). Wensink and Witholt (1981) reported that *E. coli* OMVs contained only 35% “free” lipoprotein and almost no “bound” lipoprotein. Several studies have provided supporting results to these observations, suggesting that membrane-peptidoglycan cross-linking plays a role in OMV formation in Gram-negative bacteria (Figure 1). For example, in *E. coli*, a lack of either Lpp or the L,D-transpeptidases YcfS, YbiS, and ErfK resulted in increased MV production compared with that in a wild type strain (Schwechheimer et al., 2013). In *Salmonella typhimurium*, a lack of Lpp or a mutation in the C-terminal lysine of Lpp also increased MV production (Deatherage et al., 2009). In *Neisseria meningitidis*, several proteins anchored to the outer or inner membrane through peptidoglycan, such as the pilus pore PilQ, peptidoglycan-binding protein RmpM, and the multidrug efflux pump channel protein MtrE, were less abundant in OMVs than in the outer membrane (Lappann et al., 2013).

In addition to the above proteins, OmpA is thought to be anchored to peptidoglycan through a non-covalent interaction with diaminopimelic acid, which cross-links two peptide stems to the peptidoglycan of Gram-negative bacteria (Smith et al., 2007; Park et al., 2012). Notably, a lack of OmpA also leads to increased OMV production in various Gram-negative bacteria (Sonntag et al., 1978; Song et al., 2008; Deatherage et al., 2009). These observations provide evidence for the following model of OMV formation: depletion of certain cell envelope-associated proteins, such as Lpp and OmpA, at a specific site in the outer membrane weakens outer membrane-peptidoglycan cross-linking and promotes blebbing of the outer membrane and subsequent OMV formation (Figure 1). Lpp and OmpA can be downregulated by activation of σ^*E*^, suggesting that Gram-negative bacteria may modulate OMV production in response to the accumulation of misfolded outer membrane proteins in the cell envelope (Schwechheimer et al., 2013).

Further studies showed that the accumulation of misfolded proteins in the periplasm is involved in OMV formation. In *E. coli*, the periplasmic sensor protease DegS binds to the exposed peptides of misfolded outer membrane proteins (OMPs) and initiates signal transduction through the σ^*E*^ pathway upon cell envelope stress. The dual function protease/chaperone DegP is regulated by σ^*E*^ and prevents the accumulation of misfolded proteins in the periplasm. A lack of DegP leads to increased OMV production in a temperature-dependent manner (McBroom and Kuehn, 2007). In addition, when an OMP sequence-fused cytochrome was expressed that is misfolded and accumulates in the periplasm, the chimeric protein was enriched in OMVs compared to a control periplasm protein (McBroom and Kuehn, 2007). Based on this finding and those of related studies (McBroom et al., 2006; Schwechheimer and Kuehn, 2013), it has been proposed that the accumulation of misfolded proteins, such as OMPs, expands the physical distance between the outer membrane and peptidoglycan, leading to OMV formation, which releases these toxic components into the extracellular space (Figure 1).

Several proteomic analyses of MVs that were regarded as OMVs showed that inner membrane proteins and cytoplasmic proteins are also abundant in MVs. These results were often thought to be contaminating cell fragments or debris, such as protein aggregates. Still, a large proportion of cytoplasmic proteins are frequently detected in carefully purified MV fractions (Berleman et al., 2014; Kulkami et al., 2014; Oliver et al., 2017). To explain this, Kadurugamuwa and Beveridge suggested that localized and transient breakage in the peptidoglycan, catalyzed by autolysin, leads to the formation of OMVs containing inner and outer membrane components and cytoplasmic materials in *Pseudomonas aeruginosa* (Kadurugamuwa and Beveridge, 1995; Clarke, 2018). Eighteen years later, another group showed clear images of double-bilayer OMVs from *Shewanella vesiculosa*, which were called outer-inner membrane vesicles (O-IMVs) (Pérez-Cruz et al., 2013). Although the O-IMVs were estimated to represent only 0.1% of the total MVs in this bacterium under the condition used, proteomic analyses identified some cytoplasmic proteins in the purified MV fraction (Pérez-Cruz et al., 2013). A more recent study showed that expression of an endolysin encoded by a prophage triggers MV formation in *P. aeruginosa* (Turnbull et al., 2016; Toyofuku et al., 2019). In the proposed mechanism, the cell wall is degraded by endolysin, which triggers explosive cell lysis, causing the fragmentated membrane to round up and form MVs (Figure 1). During this process, nearby intracellular components, such as DNA, become trapped in the MVs (Turnbull et al., 2016; Toyofuku et al., 2019). Explosive cell lysis triggered by DNA damage can lead to MV formation in biofilms and under anoxic conditions (Toyofuku et al., 2014; Florez et al., 2017; Cooke et al., 2019). In addition, a similar route of MV formation has been suggested in *Stenotrophomonas maltophilia*, as production of O-IMVs and phages was detected in response to ciprofloxacin stress (Devos et al., 2017). The involvement of cell lysis in MV formation would explain why inner membrane and cytoplasmic components are detected in many proteomic analyses of OMV fractions (Kulkami et al., 2014; Olaya-Abril et al., 2014; Oliver et al., 2017; Avila-Calderón et al., 2018; Taheri et al., 2018).

### Lipid Composition of MVs From Gram-Negative Bacteria

Lipidomics has recently attracted much interest as it provides fundamental information about the biochemical properties and the structural bases of MVs (Lynch and Alegado, 2017). Lipid analyses are performed using various chromatography and mass spectrometry techniques, focusing on the structures of polar head groups, chain length, and saturation of fatty acid moieties. Typically, phosphoglycerolipids (PLs) are the most abundant lipids in MVs, and numerous other lipids, including glycerolipids and lipopolysaccharides (LPS), have also been detected in MVs by lipidomic analyses. As described above, Hoekstra et al. (1976) performed lipid analyses of *E. coli* OMVs and found that their lipid composition and phospholipid/protein ratio were similar to those of the outer membrane. However, the ratio of unsaturated fatty acids to cyclopropane fatty acids was significantly higher in OMVs (1.56) than in the outer membrane (0.19). Since unsaturated fatty acids are substituted by cyclopropane fatty acids when cells enter stationary phase, the authors suggested that these OMVs were released from cells during exponential phase (Hoekstra et al., 1976).

The structures of the fatty acid moieties are often suggested to be important factors in the biogenesis of MVs because these fatty acids affect the rigidity and fluidity of the lipid membrane. Several studies have analyzed the saturation of fatty acids in MVs and the outer membrane and discussed its significance in MV biogenesis. For example, the ratio of saturated fatty acids to unsaturated and/or branched fatty acids in OMVs was higher than that in the outer membrane of *P. aeruginosa* (Tashiro et al., 2011) and *Prochlorococcus* MED4 (Biller et al., 2014), while opposite results were obtained in *Pseudomonas syringae* (Kulkami et al., 2014). In some cases, fatty acid saturation was not significantly different between OMVs and the outer membrane (Fulsunder et al., 2014; Resch et al., 2016). More direct evidence was shown in *Shewanella livingstonensis*, where depletion of a branched fatty acid, eicosapentaenoic acid (EPA), increased MV production (Yokoyama et al., 2017). EPA was also involved in the folding of the outer membrane proteins in this bacterium (Dai et al., 2012), and may influence MV formation through the accumulation of misfolded proteins.

In addition to fatty acid moieties, lipid polar head groups may play a role in MV formation, as these head groups influence lipid conformation. Phosphatidylethanolamine (PE) is a typical conical lipid that can cause membrane curvature by clustering or sequestration (Agrawal and Ramachandran, 2019). In *Haemophilus influenzae*, the PE content of OMVs from PL transporter mutants (hypervesiculation mutants), which transport PLs from the outer membrane to the inner membrane, was two-fold higher than that of wild type (Roier et al., 2016). Differences in PE content between MVs and the outer membrane have also been reported in *P. aeruginosa* (Tashiro et al., 2011). Local and asymmetrical PE accumulation or depletion in the membrane leaflet may cause structural changes in the lipid membrane that ultimately lead to blebbing of the membrane to form MVs. Interestingly, another cone-shaped lipid molecule, deacylated LPS, has been shown to induce MV production when accumulated in the membrane (Elhenawy et al., 2016).

### Composition of MVs From Gram-Positive Bacteria and Mycolic Acid-Containing Bacteria

Compared to Gram-negative bacteria, little is known about the biogenesis and composition of MVs in Gram-positive bacteria (Brown et al., 2015; Liu et al., 2018). Given that Gram-positive bacteria lack an outer membrane in their cell envelope, an important question in MV biogenesis is how the vesicles pass through the thick peptidoglycan layer.

There are several hypotheses regarding the biogenesis of MVs in Gram-positive bacteria (reviewed in Brown et al., 2015; Toyofuku et al., 2019). Notably, cell wall modification is thought to be a key process in MV formation in these bacteria. For example, endolysin is involved in MV formation by *Bacillus subtilis* (Toyofuku et al., 2017a, 2019). In contrast to the explosive cell lysis induced by the action of endolysin in *P. aeruginosa* cells (Turnbull et al., 2016), the cytoplasmic membrane of *B. subtilis* cells protrudes through the holes in the peptidoglycan that are formed by the endolysin while the cell morphology remain intact (Toyofuku et al., 2017a, 2019) (Figure 1). Endolysin-triggered MV formation has also been demonstrated in another Gram-positive bacterium, *Staphylococcus aureus* (Andreoni et al., 2019). Another peptidoglycan-hydrolyzing enzyme, autolysin, has been suggested to induce MV formation in *S. aureus* further indicating that cell wall damage is a key step in MV formation in Gram-positive bacteria (Wang et al., 2018). In this study, proteomic analysis showed that autolysins were present in MVs. Gene deletion experiments further showed that autolysins, such as Sle1, facilitate MV release by hydrolyzing peptidoglycan, particularly at sites of active cell division (Wang et al., 2018).

Several studies have focused on the lipid compositions of MVs from Gram-positive bacteria. Resch et al. (2016) reported the accumulation of phosphatidylglycerol (PG) and the depletion of cardiolipin (CL) in MVs isolated from *Streptococcus pyogenes* culture. It can be assumed that the accumulation of cylindrical lipids, such as PG, and the depletion of conical lipids, such as CL, lead to MV formation in this bacterium; however, how these MVs pass through the cell wall is unclear. In addition, MVs from *Propionibacterium acnes* possessed a remarkedly reduced amount of triacylglycerol (TG) compared to the cell membrane (Jeon et al., 2018), suggesting that the biochemical and physical properties of these MVs may be largely different from those of the cell membrane.

In addition to typical Gram-positive bacteria, there is evidence that mycolic acid-containing bacteria also produce MVs (Marsollier et al., 2007; Prados-Rosales et al., 2011; Theresia et al., 2018; Chiplunkar et al., 2019) (Figure 1). These bacteria include clinically and industrially important *Mycobacterium* and *Corynebacterium* species and are characterized by unique cell envelope structures that contain mycolic acid-containing outer membrane. Prados-Rosales et al. (2011) reported seven *Mycobacterium* species that produce MVs. In the study, they performed proteomic analyses of MVs from *Mycobacterium bovis* Bacillus Calmette-Guérin (BCG), *Mycobacterium tuberculosis* H37Rv, and *Mycobacterium smegmatis*. These analyses revealed that MVs from BCG and *M. tuberculosis* H37Rv were enriched in lipoproteins, including well-known TLR2 ligands, whereas no lipoproteins were detected in *M. smegmatis* MVs (Prados-Rosales et al., 2011). In addition, the total extractable lipids in BCG MVs predominantly consisted of polar lipids, such as PE and diacylated phosphatidylinositol dimannoside (Ac_2_PIM_2_), while mycolic acid esters were not detected in the extracted lipids (Prados-Rosales et al., 2011). Given that mycolic acid esters are major lipids in the outer membrane of *Mycobacteria*, it is possible that these MVs may originate from the inner membrane (Prados-Rosales et al., 2011) (Figure 1). Prados-Rosales and colleagues also reported that the composition of MVs from *M. tuberculosis* is influenced by iron availability. Under iron-deficient conditions, acylated glycerides and PE were enriched in the MVs, whereas acyl trehalose, an important mycobacterial cell wall component, was more abundant in MVs produced under iron-sufficient conditions (Prados-Rosales et al., 2014; Rodriguez and Prados-Rosales, 2016). Interestingly, Rath et al. (2013) reported that a cytosolic membrane-associated protein, VirR, controls MV production and cargo selection in *M. tuberculosis* (Rodriguez and Prados-Rosales, 2016). VirR contains a disordered domain, suggestive of a binding partner, in its N-terminus and was found to be associated with several proteins, including lipoproteins (Rath et al., 2013). Notably, the VirR C-terminus has a LytR family transcriptional regulator domain that plays important roles in the formation and maintenance of the cell envelope (Brown et al., 2015).

*Corynebacterium*, another genus of mycolic acid-containing bacteria, also produces MVs. Theresia et al. (2018) reported that EGTA, a calcium chelator, induced MV production by *Corynebacterium glutamicum*. CspB, a major protein component of the para-crystalline surface layer (S-layer) of the bacterium, was predominantly detected in these MVs. The authors proposed that depletion of calcium ions altered the integrity of the S-layer and subsequently triggered the release of CspB-containing MVs (Theresia et al., 2018) (Figure 1). Other proteins detected in these MVs included CspA, CmytC, and CmytB, which are cell envelope-associated proteins (Theresia et al., 2018).

Although the mechanisms underlying MV formation in *Mycobacteria* and *Corynebacteria* are still unclear, identification of additional regulatory proteins and vesiculation-inducing factors should provide clues as to how MVs are formed and released beyond the complex cell envelope of these mycolic acid-containing bacteria.

## What Determines MV Composition?

Many biochemical analyses have revealed that certain proteins and lipids are selectively accumulated in MVs through their biogenesis. Although how these molecules are selected is largely unknown, here we describe recent observations concerning the mechanisms underlying their selection.

As described above, the accumulation of misfolded proteins in OMVs has been reported in Gram-negative bacteria, suggesting that, in some cases, OMV cargo selection is a consequence of the cell envelope stress response (McBroom and Kuehn, 2007; Olofsson et al., 2010; Schwechheimer and Kuehn, 2013). Additionally, Bonnington and Kuehn (2016) reported that the LPS composition of OMVs from *Salmonella enterica* changed in response to various stresses, such as low pH. The authors hypothesized that these cells may use OMV formation as a way to selectively remove environmentally disadvantageous LPS species from the outer membrane under certain conditions. Another study showed that the size and lipid composition of *Klebsiella pneumoniae* OMVs was altered after polymyxin treatment, suggesting that the lipid composition of OMVs reflects the outer membrane remodeling associated with cell envelope stress induced by polymyxin (Jasim et al., 2018). In contrast, several studies have suggested a mechanism in which certain proteins determine the MV composition. In *Vibrio cholerae*, proteomic analysis revealed that DegP was present in the MVs (Altindis et al., 2014). Interestingly, this study demonstrated the importance of DegP in the incorporation of at least nine proteins into OMVs (Altindis et al., 2014). The authors suggested that DegP can control the protein composition of OMVs by acting as a chaperone for certain proteins. Although the determinants of protein composition in bacterial MVs are still largely unknown, it is possible that the Bam complex, which catalyzes the assembly of outer membrane proteins, may, in part, determine protein cargo selection (Bonnington and Kuehn, 2014; Hussain and Bernstein, 2018).

Haurat et al. (2011) reported that anionic LPS (A-LPS) plays a critical role in OMV protein cargo selection in *Porphyromonas gingivalis*. In this study, gingipains and TonB-dependent outer membrane proteins were excluded from OMVs in the absence of A-LPS. Yokoyama et al. (2017) reported another example of lipid-dependent cargo selection, showing that a lack of EPA altered the protein composition of OMVs. In addition, the VacJ/Yrb lipid transporter system has been suggested to be involved in phospholipid accumulation in the outer leaflet of the outer membrane and the consequent OMV formation in *H. influenzae* (Roier et al., 2016).

Although the mechanism underlying MV cargo selection in *Bacteroides fragilis* is still unknown, it is noteworthy that most of the OMV-exclusive proteins were acidic hydrolases, whereas alkaline proteins were mainly found in the outer membrane (Elhenawy et al., 2014). The authors also demonstrated that an acidic hydrolase from another *Bacteroides* bacterium was heterologously expressed and selectively packed in *B. fragilis* OMVs (Elhenawy et al., 2014). Therefore, at least in some *Bacteroides* species, there may be an interesting mechanism in which the cargo proteins are selectively packed into OMVs based on their function or biochemical properties, such as pI.

Another interesting observation is a correlation between OMV size and protein contents (Turner et al., 2018). The authors found less protein content and diversity in small *Helicobacter pylori* OMVs (20–100 nm) than in larger OMVs (90–450 nm). These OMV may have originated from different formation routes leading to different protein contents.

## Composition and Action Mechanisms of OMVs

Membrane vesicles play important roles in bacteria-host interactions. Bacterial MVs often have immunomodulating activities in host animals due to the presence of numerous molecules with microorganism-associated molecular patterns (MAMPs), including DNA, RNA, lipoproteins, LPS, and peptidoglycan (Kaparakis-Liaskos and Ferrero, 2015; Tan et al., 2018; Wang et al., 2019). For this reason, MVs have been intensively investigated for their potential in the development as novel vaccine platforms. Diverse hydrolytic enzymes are contained in the MVs of pathogens, suggesting that they may act in infection processes, such as the invasion of epithelial barriers (Olofsson et al., 2010; Lappann et al., 2013; Liu et al., 2018; Zarzecka et al., 2019). In addition, proteins involved in biofilm formation have been detected in MVs, suggest their potential role in biofilm formation and pathogen colonization in host animals (Altindis et al., 2014; Wagner et al., 2018).

**FIGURE 2:**
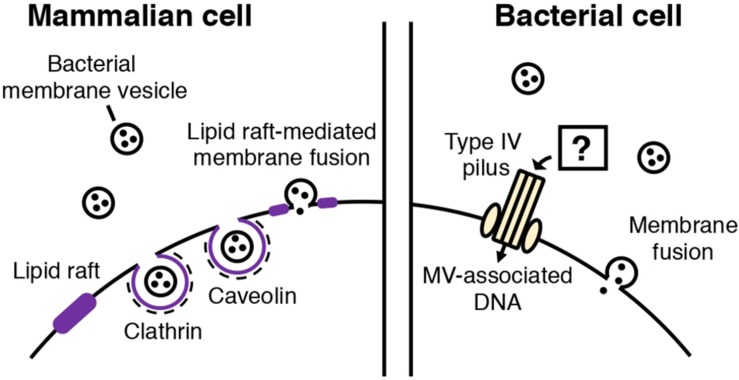
Routes of MV entry into mammalian and bacterial cells. In mammalian cells, bacterial MVs are thought to be internalized through several routes. Cholesterol-rich lipid rafts in the plasma membrane of the mammalian cell mediates MV entry through caveolin-mediated endocytosis or fusion of the lipid raft and bacterial MV. Clathrin-mediated endocytosis is also involved in MV internalization. In bacterial cells, two types of MV entry have been proposed: MV components, such as DNA, may be internalized into bacterial cells through type IV pili, or MVs may also fuse with the cellular membrane of the bacterial cell, depending on the membrane composition.

In MV-dependent immunomodulation, the attachment and uptake of MVs by host cells has been proposed as an initial step (Kaparakis-Liaskos and Ferrero, 2015; Tan et al., 2018; Wang et al., 2019). So far, it is proposed that bacterial MVs are taken up by mammalian host cells through similar routes as other extracellular vesicles (EVs) (Figure 2). In mammalian cells, EVs (originating from mammalian cells) are taken up by recipient cells through phagocytosis or clathrin, caveolin-mediated endocytosis (Mulcahy et al., 2014). There is also evidence showing that lipid rafts are involved in EV uptake (Mulcahy et al., 2014). In addition, EVs may also fuse with the plasma membrane or be internalized via macropinocytosis, during which the EVs are enclosed into the lumen of macropinosomes or caught in membrane raffles before entry (Mulcahy et al., 2014).

Schaar et al. (2011) reported that *Moraxella catarrhalis* OMVs enter host cells by binding to lipid rafts associated with caveolin, after interaction with Toll-like receptor 2 (TLR2). The authors also identified the adhesins and virulence factors responsible for triggering the immune response (Schaar et al., 2011). Several studies have also shown that specific, potent inhibitors of endocytosis decrease MV entry into host cells (Kesty et al., 2004; Bomberger et al., 2009; Furuta et al., 2009; Parker et al., 2010; Schaar et al., 2011; Crowley et al., 2013; O’Donoghue et al., 2017). MV fusion with the host cell membranes has been shown (Kesty et al., 2004). It was also suggested that the route of cellular entry may vary according to the MV composition and size. Parker et al. (2010) showed that *H. pylori* OMVs are internalized via clathrin-mediated endocytosis in the absence of VacA toxin, while VacA^+^ OMVs can be taken up through several routes. Turner et al. (2018) reported that smaller *H. pylori* OMVs (20–100 nm) preferentially entered host cells via caveolin-mediated endocytosis instead of clathrin-mediated endocytosis, while larger OMVs (90–450 nm) entered via several pathways including endocytosis. Based on the result that OMV size appears to be correlated with their protein composition, the authors proposed that the difference in the mechanisms of OMV entry may reflect the variation in OMV size and protein composition. O’Donoghue et al. (2017) showed that the LPS composition of an OMV determines major route and kinetics of host cell entry. OMVs lacking O-antigen are internalized via clathrin-mediated endocytosis, while in the presence of O-antigen, OMVs are likely to enter via lipid raft-dependent and receptor-independent routes (O’Donoghue et al., 2017).

Compared to MV entry into mammalian cells, MV uptake by bacterial cells is poorly understood. Although the entry of the membranous components or cargo into bacterial cells is an important step for MV function (Klieve et al., 2005; Mashburn and Whiteley, 2005; Domingues and Nielsen, 2017; Tashiro et al., 2017; Toyofuku et al., 2017b), the underlying mechanisms are unclear.

Fulsunder et al. (2014) showed that MVs mediate horizontal gene transfer between *E. coli* and *Acinetobacter baylyi*. In their experiments, a plasmid harboring an antibiotic resistance gene was transferred from one bacterium to the other via OMVs. They also demonstrated that OMVs were attached to or internalized by the recipient cells via transmission electron microscopy using immunogold-labeled OMVs. Importantly, the authors showed that the competence proteins of *A. baylyi*, such as ComA and ComB, play a role in the uptake of DNA delivered by OMVs. Therefore, it has been suggested that OMVs are lysed upon contact with the outer membrane of the bacterium, followed by type IV pilus-mediated transport of DNA (Fulsunder et al., 2014).

It has also been proposed that MVs transport their contents into bacterial cells through membranous fusion (Kadurugamuwa and Beveridge, 1996; Kim et al., 2016; Tashiro et al., 2017). For the membranes to fuse, they need to come into contact. Reducing intermembrane hydration repulsion, decreasing bilayer surface density or polarity, and increasing the hydrophobicity of the intermembrane hydrophilic region are known to bring two membranes in close contact (Ohki, 1982; Cevc et al., 1985; Rand and Parsegian, 1989; Burgess et al., 1992; Mondal Roy and Sarker, 2011). Kadurugamuwa suggested that two divalent cations, Mg^2+^ and Ca^2+^, which form salt bridges between MVs and the outer membrane, initiate membrane fusion and deliver the autolysin cargo to the cell (Kadurugamuwa and Beveridge, 1996; Wang et al., 2016; Oshima and Sumitomo, 2017). Tashiro and colleagues showed that *Buttiauxella agrestis* MVs selectively interact with *Buttiauxella* species. Based on Derjaguin-Landau-Verwey-Overbeek (DLVO) theory and physicochemical analyses, they suggested that van der Waal’s forces and electric repulsion energy are involved in the selective interaction of the bacteria with MVs (Tashiro et al., 2017).

Given that bacterial MVs can fuse with the lipid rafts in eukaryotic cells, microdomains in bacterial cells may also function as the contact sites for MVs. Functional microdomains in the membrane that contain certain lipids, such as PE, CL, diacylglycerols, cholesterols, or polyisoprenoids, have been suggested in various bacteria, including both Gram-negative and Gram-positive species (Matsumoto et al., 2006; LaRocca et al., 2010, 2013; López and Kolter, 2010; Toledo et al., 2018) although their compositions and structural bases remain largely unknown. PE and CL, which are inverted hexagonal phase-forming lipids, are major components of bacterial MVs, and the transition from the lamellar bilayer phase to the inverted hexagonal phase could facilitate the merging of lipids required for membrane fusion, once MVs come in contact with the cell (Powell and Marsh, 1985; Lewis and McElhaney, 1993; Kinnunen, 1996; Mondal Roy and Sarker, 2011). Some proteins, such as dynamin-like protein DynA, that mediate lipid mixing (Guo and Bramkamp, 2019), may also be involved in vesicle fusion, and this requires further investigations.

## Conclusion

As we have reviewed here, biochemical approaches are powerful tools for elucidating the composition, biogenesis, and function of MVs. Proteomic and lipidomic analyses indicate that the composition of MVs depends on the growth conditions (Choi et al., 2014; Kim et al., 2016; Wagner et al., 2018; Taboada et al., 2019), suggesting that the MVs are heterogenous and their functions differ depending on the environment. On the other hand, recent studies have shown different pathways of MV formation, which lead to different MV compositions. Given this heterogeneity, we need to develop methods that enable separation and analysis of different types of MVs in cell culture as well as natural settings and body fluids. Currently, most MVs are isolated and purified by density-gradient ultracentrifugation or gel-filtration chromatography in which the particles are separated depending on their density or size in liquid solutions. Although these procedures can exclude the major contaminants (such as flagella and protein aggregates) from the purified MV solution, other techniques separating and sorting MVs depending on different properties, such surface charges, are required to investigate more precisely the biochemical properties of each MVs produced via different routes. To fully understand the true functions of each MV particles, we also need to understand the molecular mechanisms of how each types of MVs deliver their cargos to the target cells. Integrating biochemical information with imaging techniques and molecular biological approaches, may help us tackle such challenges.

## Author Contributions

All authors listed have made a substantial, direct and intellectual contribution to the work, and approved it for publication.

## Conflict of Interest

The authors declare that the research was conducted in the absence of any commercial or financial relationships that could be construed as a potential conflict of interest.
